# Shear Stress Induced by an Interstitial Level of Slow Flow Increases the Osteogenic Differentiation of Mesenchymal Stem Cells through TAZ Activation

**DOI:** 10.1371/journal.pone.0092427

**Published:** 2014-03-21

**Authors:** Kyung Min Kim, Yoon Jung Choi, Jun-Ha Hwang, A. Rum Kim, Hang Jun Cho, Eun Sook Hwang, Joong Yull Park, Sang-Hoon Lee, Jeong-Ho Hong

**Affiliations:** 1 Division of Life Sciences, Korea University, Seoul, Republic of Korea; 2 Department of Biomedical Engineering, Korea University, Seoul, Republic of Korea; 3 College of Pharmacy and Global Top5 Research Program, Ewha Womans University, Seoul, Republic of Korea; 4 School of Mechanical Engineering, College of Engineering, Chung-Ang University, Seoul, Republic of Korea; University of Illinois at Chicago, United States of America

## Abstract

Shear stress activates cellular signaling involved in cellular proliferation, differentiation, and migration. However, the mechanisms of mesenchymal stem cell (MSC) differentiation under interstitial flow are not fully understood. Here, we show the increased osteogenic differentiation of MSCs under exposure to constant, extremely low shear stress created by osmotic pressure-induced flow in a microfluidic chip. The interstitial level of shear stress in the proposed microfluidic system stimulated nuclear localization of TAZ (transcriptional coactivator with PDZ-binding motif), a transcriptional modulator of MSCs, activated TAZ target genes such as *CTGF* and *Cyr61*, and induced osteogenic differentiation. TAZ-depleted cells showed defects in shear stress-induced osteogenic differentiation. In shear stress induced cellular signaling, Rho signaling pathway was important forthe nuclear localization of TAZ. Taken together, these results suggest that TAZ is an important mediator of interstitial flow-driven shear stress signaling in osteoblast differentiation of MSCs.

## Introduction

Cellular architectures sense extracellular mechanical stimuli and transduce them into intracellular signals that affect cell growth, differentiation, and migration. Several components of mechanotransduction signaling eventually activate the transcription of target genes. Among these components, TAZ (transcriptional coactivator with PDZ-binding motif) and YAP (Yes associated protein) were recently characterized as signaling mediators of mechanotransduction [1], [2]. TAZ and YAP are transcriptional co-regulators identified as 14-3-3-binding proteins [3]. For transcriptional regulation, TAZ and YAP interact with many transcription factors, including Runx2, TEADs, TTF-1/Nkx2.1, Tbx5, Pax3, Smad2/3-4 complexes, and MyoD [4]–[12]. These interactions activate or suppress several target genes in a context-dependent manner, thereby producing diverse biological functions. Functionally, TAZ and YAP have been characterized as effector proteins in the Hippo signaling pathway, which plays an important role in cell proliferation, tumorigenesis, and stem cell self-renewal [13], [14]. TAZ also modulates mesenchymal stem cell (MSC) differentiation by activating osteoblast and myoblast differentiation and inhibiting adipocyte differentiation [4], [12]. TAZ stimulates Runx2-mediated gene transcription, but its interaction with PPARγ inhibits PPARγ-mediated gene transcription [4]. Although the role of TAZ in MSC differentiation has been established, the regulatory signal of differentiation is not clearly understood.

Interstitial flow is the extremely slow flow of extravascular fluid through a three-dimensional matrix. It produces a low shear stress because of the high flow resistance of the extracellular matrix [15]. Such extremely slow flows (shear stress of ≤ 0.01 Pa) are not only physiological but also expected to have roles in cell morphogenesis and pathogenesis [16]. Functionally, interstitial flow affects embryonic development and maintains tissue function in muscle, cartilage, and bone [15], [17]. MSCs in bone marrow are targets for interstitial flow, and the shear stress induced by the flow regulates cell proliferation and differentiation [18], [19]. Although interstitial flow in bone has not been clearly reported elsewhere, it has attracted the interest of scientists because interstitial flow in bone seems to trigger a signal transduction process for bone remodeling and bone formation [20].

Experimental platforms to analyze shear stress-mediated cellular signaling in a small population of cells have been developed, but they need bulky apparatus to produce the signal [21], [22]. We previously made a novel microfluidic platform to generate a concentration gradient without the assistance of bulky peripheral devices [23], [24]. The unique part of this system was an osmosis-driven pump fabricated with a polydimethylsiloxane (PDMS) chamber, a semi-porous membrane (cellulose membrane), and polyethylene glycol (PEG), which was used as the driving agent for osmosis. The shear stress driven by osmotic pressure ranged in magnitude down to the *in vivo* interstitial shear stress level; it was maintained long-term (days to weeks). Minimal operator's intervention and no need for an external power source provided great advantages such as reproducibility, controllability, and stability for studying shear stress-driven cellular biological functions.

In the present study, we investigated the interstitial flow level of shear stress-induced osteogenic differentiation using an osmotic pump equipped with a microfluidic chip. The flow rates generated by the osmotic pump were accurately controlled and stably maintained without any external electrical power sources. The slow flows produced were not only physiological, comparable to those of interstitial flow, but also consumed a minimal amount of expensive reagents, such that quantitative cell analyses were easily achieved. Our results show that TAZ, a transcriptional coregulator of MSC, plays an important role in low shear stress-induced osteogenesis. Moreover, the osmotic pump-driven microfluidic chip provides a useful system for the study of interstitial flow-induced cellular mechanisms.

## Materials and Methods

### Chip fabrication and simulation

**Figure 1:**
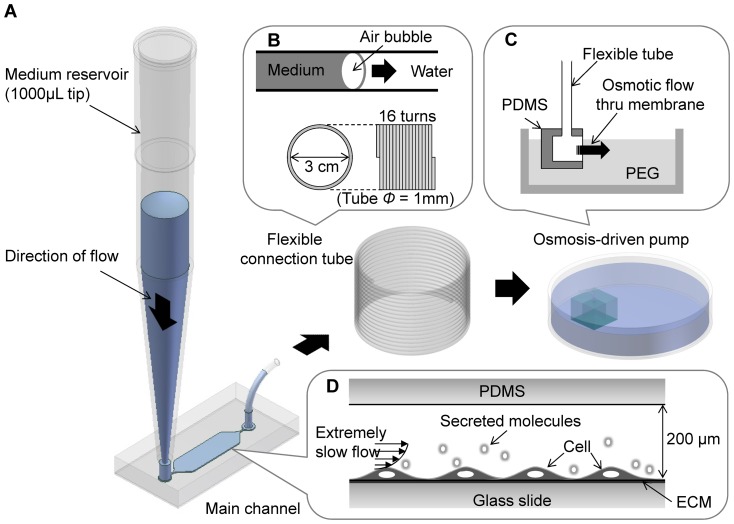
Experimental system. (A) The system consisted of an inlet reservoir (1000-μL pipette tip), a microfluidic channel where cells were cultured and exposed to shear flow, a coiled tube, and an osmotic pump dipped into a Petri dish containing 0.1 M PEG solution. (B) The coiled tube had a large capacity that served as an outlet reservoir providing clean water to the osmotic pump. An air bubble moved through the tube to prevent the mixing of medium and water. (C) The osmotic flow was generated at the membrane window by the concentration difference between water and PEG solution. (D) Cells in the main microchannel (200-μm height) were exposed to an extremely slow interstitial level of flow.

The osmotic pump-harnessed microfluidic chip and operational scheme are illustrated in Figure 1. The main microchip channel was 4 mm wide, 10 mm long, and 200 μm high and was connected to a 1000-μL micropipette tip (Figure 1A), which was used as an inlet reservoir. The fluid flow through the main channel reached to the outlet tubing in which flow was separated by an air bubble (Figure 1B), thus preventing the unwanted mixing of medium (containing cell-secreted molecules and wastes) and clean water (pure H_2_O for proper osmotic flow). The tubing was connected to the osmotic pump unit, which was submerged in polyethylene glycol (PEG) solution (Figure 1C). The osmotic pump unit was a simple PDMS cubic chamber with dimensions of 1 cm × 1 cm × 1 cm and a window made of cellulose membrane (5 mm × 5 mm) [23]. Osmotic flow through the cellulose membrane occurred because of the solute differential produced when the inner chamber was filled with water and the outer chamber was filled with 0.1 M PEG solution (molecular weight  =  2000); the osmotic flow generated by the 0.1 M PEG solution was approximately 0.274 μL/min [23]. The flow path of the whole system was single-inlet–single-outlet, and the cells growing on the main channel were exposed to a steady and slow osmosis-driven flow (Figure 1D) without the need for peripheral electrical devices.

Due to the difficulties of measuring fluid dynamic variables (velocity profiles, pressure distribution, etc.) of the flow in the main channel, a three-dimensional computer simulation was performed to analyze the flow in the microfluidic channel by using a finite-volume method-based commercial code, FLUENT 5.5 (Fluent, USA). Because the flow was laminar and steady in the microchannel, the Navier–Stokes equations and the mass conservation equation were calculated. The fluid was assumed as a homogeneous, incompressible Newtonian fluid. The density of the culture medium was assumed to be same as that of water, 993.2 kg/m^3^, and the dynamic viscosity was 0.000785 kg/m·s at 37°C [25], [26]. The boundary conditions were defined based on the inlet conditions for the flow rate (0.274 μL/min) in the inlet port and the outflow conditions for the outlet. The convergence of computation was achieved when the residuals of the momentum and conservation equations reached 10^−6^.

### Cell culture and osteoblast differentiation

Human MSCs, purchased from Lonza, were maintained in Dulbecco's modified Eagle's medium (DMEM) supplemented with 10% fetal bovine serum (HyClone) and antibiotics (100 units/mL penicillin, 100 μg/mL streptomycin). Microfluidic chips were coated with poly-l-lysine (0.01%). MSCs (1.5 × 10^5^ cells/mL in a 200 μL suspension) were then loaded into the main channel. After waiting 2 h for cell attachment to the chips, the osmotic pump started to generate the flow and shear stress in the main channel by using the 0.1 M PEG solution. As a negative control, the osmotic pump was not operated. For osteogenic differentiation, at confluent conditions the culture medium was changed to the differentiation media (DMEM containing 50 μg/mL ascorbic acid, 10 mM β-glycerophosphate, 0.5 μM dexamethasone, and 10% FBS (Fetal Bovine Serum)) for 4 days. The differentiation medium was replaced every 2 days.

### Stable cell lines

Phoenix cells were cultured in 100-mm dishes and transfected using the calcium phosphate method with pLL3.7 (vector control) and pLL3.7-TAZ (Ti), in which the sequence for the sense oligonucleotide was 5′-AGGTACTTCCTCAATCACA-3′ [27]. Viral supernatants were harvested 24 h after transfection and applied to MSCs in DMEM containing 10% FBS and 4 μg/mL polybrene. Twenty-four hours later, the cells were incubated with 0.5 μg/mL puromycin to eliminate uninfected cells. After 1 week of selection, cells were used in differentiation studies. TAZ depletion was verified by western blot analysis with a TAZ antibody.

### Immunoblot analysis

Cells were harvested and lysed in TNE lysis buffer containing 20 mM Tris-HCl (pH 7.5), 1% NP-40, 150 mM NaCl, 2 mM EDTA, 50 mM NaF, 1 mM Na-orthovanadate, and protease inhibitors. Total cell lysates were denatured in 4× SDS sample buffer (250 mM Tris-HCl (pH 6.8), 10% β-mercaptoethanol, 30% glycerol, 0.02% bromophenol blue, 8% SDS) and resolved in 8% SDS-PAGE gels. After transfer, the membranes were incubated with TAZ antibodies [3].

### Immunocytochemical analysis

After culturing MSCs inside the microfluidic chips, cells were fixed in 4% paraformaldehyde for 15 min at room temperature. After blocking with 0.3% Triton X-100 or with 5% bovine serum albumin in phosphate-buffered saline (PBS), the cells were probed with a TAZ-specific antibody diluted with 0.3% Triton X-100 or with 1% bovine serum albumin in PBS. After incubation with primary antibodies, the cells were washed and probed with the appropriate secondary antibodies (Pierce® Antibody) for 2 h in the dark at room temperature. Finally, fluorescent images were captured using a confocal laser-scanning microscope (Carl Zeiss LSM 700) after counterstaining with Alexa Fluor 635 (Phalloidin) and DAPI.

### Staining for alkaline phosphatase

To visualize alkaline phosphatase activity in the differentiated cells, MSCs were fixed with 3.7% formaldehyde at room temperature for 10 min and rinsed with PBS. They were then stained with 0.1 mg/mL naphthol AS-MX phosphate (Sigma N4875), 0.5% *N*,*N*-dimethylformamide (DMF, Sigma D4551), 2 mM MgCl_2_, and 0.6 mg/mL Fast Blue BB salt (Sigma F3378) in 0.1 M Tris-HCl (pH 8.8) in the dark at room temperature. After staining, the samples were rinsed with water.

### Real-time PCR analysis

Total RNA was isolated using TRIzol reagent (Gibco-BRL, Invitrogen) and reverse transcribed into cDNA. Real-time PCR reactions were performed using an ABI-Prism 7700 sequence detector (Perkin-Elmer Applied Biosystems, Foster City, CA, USA). The relative transcript levels were calculated by normalizing the threshold cycle (Ct) values to that of GAPDH. The following primers were used to analyze human gene expression: GADPH, 5′-GACCCCTTCATTGACCTCAAC-3′ and 5′-GGTGCCATGGAATTTGCCATG-3′; TAZ, 5′-CCTCTTCAATGATGTAGAGTCTGC-3′ and 5′-AGTGATTACAGCCAGGTTAGA-3′; Runx2, 5′-AGAGGTACCAGATGGGACTGT-3′ and 5′-GGTAGCTACTTGGGGAGGATT-3′; DLX5, 5′-CTACAACCGCGTCCCAAG-3′ and 5′-GCCATTCACCATTCTCACCT-3′; Msx2, 5′-CTACCCGTTCCATAGACCTGT-3′ and 5′-GAGAGGGAGAGGAAACCCTTT-3′; CTGF, 5′-CGACTGGAAGACACGTTTGG-3′ and 5′-AGGCTTGGAGATTTTGGGAG-3′; and Cyr61, 5′-GAGTGGGTCTGTGACGAGGAT-3′ and 5′-GGAGGCATAGCTGCCTTTTCC-3′.

### Statistical analysis

All data are given as mean ± SD. The results were analyzed for statistically significant differences using Student's *t*-test, and statistical significance was set at p < 0.05.

## Results

### Numerical calculation of the interstitial flow level in the microchip

**Figure 2:**
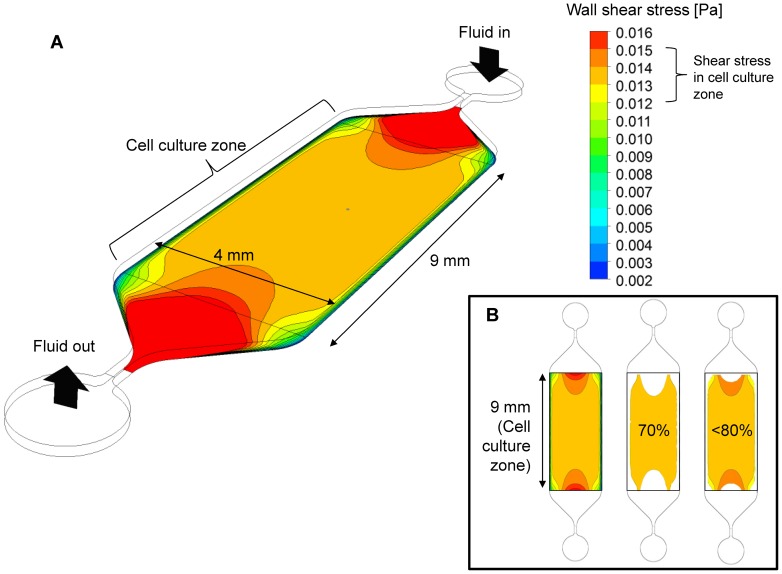
Computed shear stress distribution. (A) The rectangular area (9 mm × 4 mm) of the main microchannel was considered the cell culture zone for experimental observation. Due to the tapered geometry near the inlet and outlet ports, higher shear stresses developed in these areas, whereas almost uniform shear stress developed in the cell culture zone. (B) Quantitative analysis showed that over 70% of the cell culture zone had a good uniformity of shear stress (approximately 7% variation) and over 80% of the area had an acceptable uniformity (approximately 20% variation).

For accurate numerical prediction of wall shear stress of the interstitial level of flow created in the main channel, an appropriately high grid density was used. The mesh test was performed using a mesh with 260,000 or 100,000 nodes over the computational domain. The differences in the observed average/maximum shear stresses and pressure values were <1% between the 2 grid densities, implying that both grid systems were appropriate for the calculation, and the 260,000-node grid system was selected for calculation. Because of the channel design, a large area of uniform wall shear stress was developed in the middle region of the channel (cell culture zone) (Figure 2A). The results showed a flat shear stress profile almost everywhere except in regions near the narrow ducts connecting the inlet and outlet ports. Therefore, the cells were exposed to an almost uniform shear stress in a given cell culture zone that spanned 9 mm along and 4 mm across the channel, which can be considered the “effective uniform shear stress region.” We calculated the exact shear stress profile and found the shear stress in this region was approximately 0.0135 Pa. However, near the side walls the velocity (and thus shear stress) decreased significantly due to no slip conditions of flow at the wall. An image analysis indicated that about 71% of the cell seeding area was exposed to 0.013–0.014 Pa (∼ 7% variation) and over 80% was exposed to 0.012–0.015 Pa (variation less than 20%), which can be accepted as a reasonably uniform shear stress for our study (Figure 2B). Therefore, it was regarded that the cells cultured in the cell culture zone of the microchannel were exposed to a uniform flow and shear stress level. Velocity distribution of the channel flow was shown in Figure S1.

### Shear stress increases TAZ expression and nuclear localization

**Figure 3:**
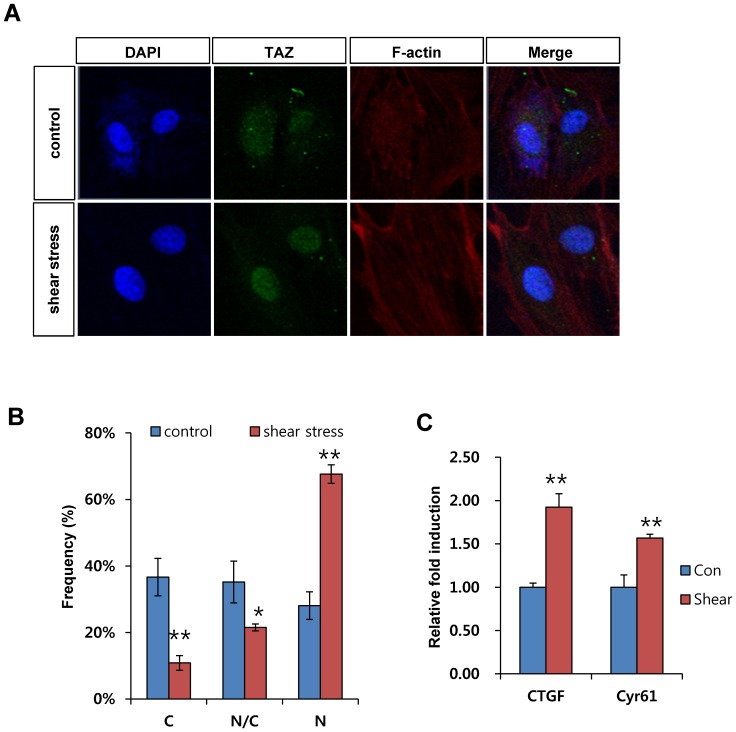
Shear stress stimulates TAZ. (A) Shear stress increases the nuclear localization of TAZ. MSCs were loaded onto the microfluidic chip, and osmotic pressure-driven shear stress was applied to the chip. Stationary states were used as controls. Media change was assessed every 12 hours for control cells. After 48 h, the cells were fixed and analyzed using immunocytochemical analysis. A TAZ-specific antibody was used to observe the location of TAZ. Scale bars indicate 20 μm size. (B) The cellular distribution of TAZ in (A) was quantitatively analyzed to determine whether it was higher in the nucleus (N > C), higher in the cytoplasm (N < C), or evenly distributed between the nucleus and cytoplasm (N  =  C). The percentage was scored after observing cells from three independent experiments of (A). * indicate p-value as determined by Student's *t*-test; * for p < 0.05, ** for p < 0.01. (C) Increased transcriptional activity of TAZ after shear stress. *CTGF* and *Cyr61* mRNA were analyzed by qRT-PCR. Their relative expression was calculated after normalization to the *GAPDH* level. ** for p < 0.01, *t*-test.

To study the role of TAZ in response to interstitial level of shear stress, the expression and localization of TAZ was analyzed in MSCs after microfluidic shear stress. TAZ expression and localization were revealed by green fluorescence signal. As shown in Figure 3A, nuclear localization of TAZ was significantly induced after shear stress. Compared to control cells, approximately 70% of cells showed increased nuclear localization of TAZ after shear stress (Figure 3B).

Next, for studying the transcriptional activity of TAZ after nuclear localization, the expression of *CTGF* and *Cyr61*, TAZ target genes, were analyzed. As shown in Figure 3C, *CTGF* and *Cyr61* mRNAs were significantly induced after shear stress by about 2 and 1.5 fold respectively, which suggests that shear stress stimulated the transcriptional activity of TAZ. Taken together, the results show that shear stress stimulates TAZ activity in MSCs.

### Shear stress stimulates osteoblast differentiation of MSCs in our microfluidic chip

**Figure 4:**
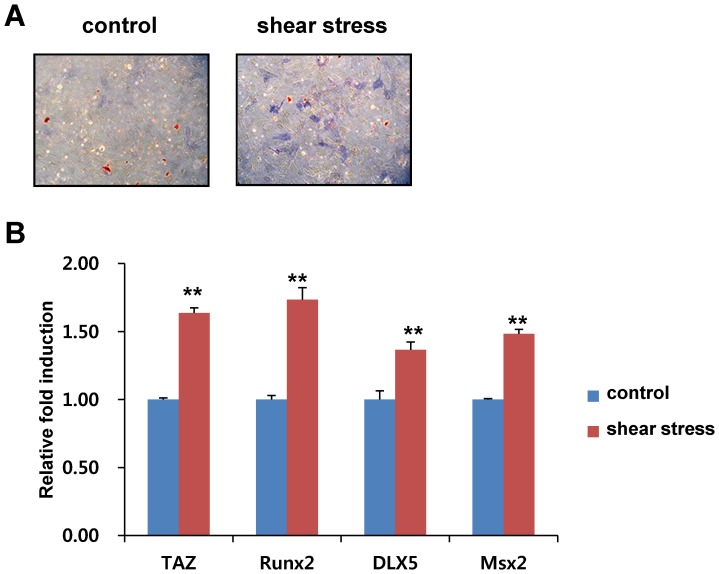
Shear stress stimulates osteoblast differentiation. (A) Increased alkaline phosphatase activity by shear stress. MSCs were loaded onto the chips and differentiated into osteoblasts in the presence of osteogenic differentiation media for 4 days. Alkaline phosphatase activity in the differentiated cells was stained according to the experimental method. Control cells were incubated in the osteogenic differentiation media without low shear stress. (B) Increased osteogenic marker gene expression by shear stress. Total RNA in (A) was harvested at 2 days after differentiation and analyzed by reverse transcription and qRT-PCR. The relative expression levels of *TAZ*, *Runx2*, *DLX5*, and *Msx2* were determined after normalization to the *GAPDH* level. ** for p < 0.01, *t*-test.

TAZ has been shown to interact with Runx2 and stimulate osteoblast differentiation of MSCs [4]. Because nuclear localized TAZ can stimulate osteogenic differentiation in the presence of osteogenic differentiation media, we studied whether microfluidic shear stress stimulated osteogenic differentiation in our microfluidic chip. For the experiment, MSCs on microfluidic chips were incubated with osteogenic differentiation media in the absence or presence of shear stress. The osteogenic differentiation potential of the cells was analyzed by alkaline phosphatase activity, a marker of osteogenic differentiation. As shown in Figure 4A, shear stress significantly induced alkaline phosphatase activity indicated by increased blue colour containg cells and intensity. To define the osteogenic potential further, total RNAs were isolated, and the expression of osteogenic differentiation marker genes *TAZ*, *Runx2*, *DLX5*, and *Msx2* were analyzed using RT-PCR. In Figure 4B, shear stress stimulated the induction of genes. We also investigated mineralization activity. As shown in Figure S2, shear stress significantly increased the mineralization evidenced by Von Kossa staining. Thus, these results suggest that an interstitial level of low shear stress stimulates osteogenic differentiation on microfluidic chips.

### Depletion of TAZ does not stimulate shear stress-induced osteogenic differentiation

**Figure 5:**
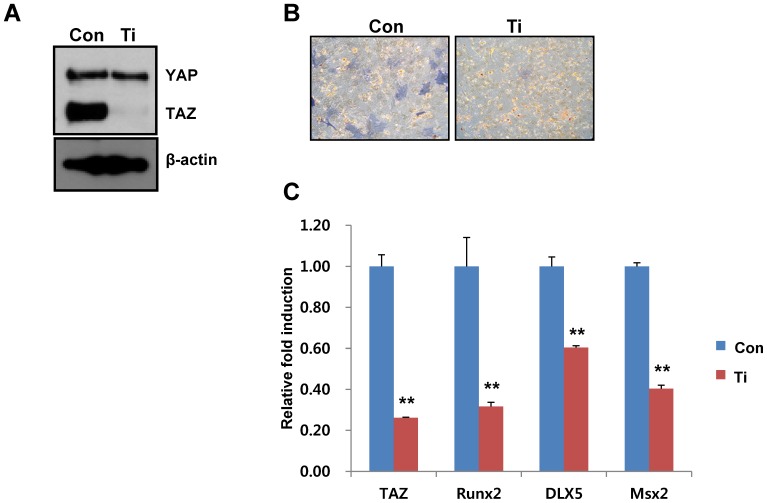
Cells with decreased TAZ expression show defects in shear stress-induced osteogenic differentiation. (A) Depletion of TAZ in MSCs was assessed by infecting cells with a retrovirus carrying TAZ small hairpin RNA. The expression of TAZ was analyzed by immunoblot analysis. Con and Ti indicate vector control cells and TAZ-depleted cells, respectively. (B) Decreased alkaline phosphatase activity in TAZ-depleted cells after shear stress. The Con and Ti cells were differentiated into osteoblasts in the presence of osteogenic differentiation media for 4 days with microfluidic shear stress. Alkaline phosphatase activity in the differentiated cells was stained according to the experimental method. (C) Decreased osteogenic marker gene expression in TAZ-depleted cells after shear stress. Total RNA in (B) was harvested at 2 days after differentiation and analyzed by reverse transcription and qRT-PCR. The relative expression levels of *TAZ*, *Runx2*, *DLX5*, and *Msx2* were determined after normalization to the *GAPDH* level. ** for p < 0.01, *t*-test.

To understand whether TAZ is important for microfluidic shear stress-induced osteogenic stimulation, TAZ-depleted cells were generated using a TAZ small hairpin RNA (shRNA)-producing retrovirus. As shown in Figure 5A, the level of TAZ was lower in the TAZ shRNA-producing cells than in the control cells. The potential for osteogenic differentiation was assessed with the control and TAZ-depleted cells. Shear stress significantly induced osteogenic differentiation in control cells, but not in TAZ-depleted cells, as indicated by the alkaline phosphatase activity (Figure 5B). Additionally, decreased expression of osteogenic marker genes was observed in TAZ-depleted cells (Figure 5C). The results show that microfluidic shear stress-induced osteogenic differentiation required TAZ, suggesting that TAZ is an important component of shear stress signaling in osteogenic differentiation of MSCs.

### Rho activation is important for nuclear localization of TAZ expression and osteogenic stimulation

**Figure 6:**
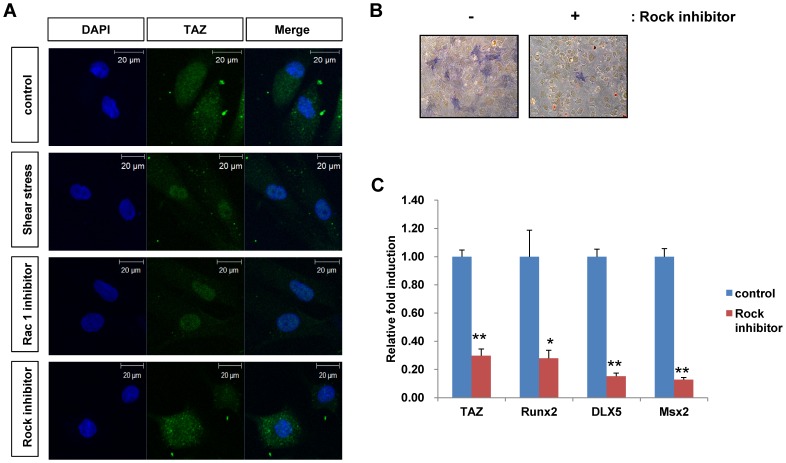
Rho GTPase is involved in TAZ localization after shear stress. (A) MSCs were loaded onto microfluidic chips, and shear stress was applied to the chips in the presence of 50 μM Y27632 (Rock inhibitor) and 100 μM Rac1 inhibitor. After 24 h, the cells were fixed and analyzed by immunocytochemistry. A TAZ-specific antibody was used to observe the location of TAZ. Scale bars indicate 20 μm size. (B) Decreased alkaline phosphatase activity in Y27632-treated cells after shear stress. Control and Y27632-treated cells were differentiated into osteoblasts in the presence of osteogenic differentiation media for 4 days with microfluidic shear stress. Alkaline phosphatase activity in the differentiated cells was determined according to the experimental method. (C) Decreased osteogenic marker gene expression in Y27632-treated cells after shear stress. Total RNA in (B) was harvested at 2 days after differentiation and analyzed by reverse transcription and qRT-PCR. The relative expression levels of *TAZ*, *Runx2*, *DLX5*, and *Msx2* were determined after normalization to the *GAPDH* level. * for p < 0.05, ** for p < 0.01, *t*-test.

Shear stress stimulates many intracellular signaling pathways. Among them, focal adhesion-mediated activation of GTPase proteins is well known for mechanotransduction [28], [29]. Thus, we tested whether GTPase proteins are involved in the nuclear localization of TAZ. For the experiments, cells were treated with Y27632 (an inhibitor of Rock, a downstream kinase of Rho signaling) and a Rac1 inhibitor. As shown in Figure 6A, shear stress-induced nuclear localization of TAZ was reduced in the presence of Rock inhibitor, but not Rac1 inhibitor, indicating that the Rho activation signal is involved in the regulation of TAZ activity. We also investigated the Rho expression after the shear stress, but we did not observe significant induction compare to control (data not shown). Next, to study whether Rho signaling regulates shear stress-induced osteogenic differentiation, cells were co-treated with Rock inhibitor during shear stress-induced osteogenic differentiation. As shown in Figure 6B, shear stress-induced osteogenic stimulation was inhibited in the presence of Rock inhibitor, as indicated by the decreased alkaline phosphatase staining. This suggests that shear stress-induced osteogenic stimulation requires the activation of the Rho signaling pathway. Expression of the osteogenic marker genes was also decreased in the presence of Rock inhibitor (Figure 6C). Thus, the results suggest that the Rho activation signal is involved in shear stress-induced osteogenic stimulation through the regulation of TAZ activity and localization.

## Discussion

Mechanical loading of bone produces fluidal shear stress and stimulates osteoblast differentiation. Here, we showed that TAZ plays an important role in osteogenic differentiation induced by shear stress. We used an osmotic pump-driven microfluidic chip that generates interstitial level of slow flow (and thus low shear stress level) to induce osteogenic differentiation of MSCs. We observed that shear stress significantly induced TAZ nuclear localization and transcriptional activity, thereby facilitating osteogenic differentiation. Thus, our results suggest that TAZ is an important signaling mediator of shear stress induced by interstitial flow.

TAZ and YAP, a paralog of TAZ, are known mediators of mechanotransduction signaling induced by extracellular matrix (ECM) stiffness [1]. Stiff ECM stimulates the nuclear localization of TAZ/YAP and facilitates osteogenic differentiation, whereas soft ECM inhibits their nuclear localization and induces adipogenic differentiation [1], [30]. Stiff ECM stimulates TAZ/YAP through the activation of Rho GTPase. Rho activation-induced F-actin polymerization activates TAZ and YAP [1]. Thus, our observations are consistent with the previous report in that shear stress stimulated the nuclear localization of TAZ through Rho activation, and microfluidic shear stress increased F-actin polymerization (Figure 3A). Treatment with a Rock inhibitor decreased the nuclear localization of TAZ (Figure 6A). Thus, our results indicate that changes in the cytoskeletal architecture induced by shear stress are important for TAZ activity in shear stress signaling. Notably, Rho activation stimulates osteogenic differentiation, but a dominant-negative form of Rho A induces adipogenesis [31]. Mechanical strain regulates the osteogenic and adipogenic differentiation of MSCs, stimulating osteogenesis and inhibiting adipogenesis [32]–[35]. Interestingly, TAZ also stimulates osteogenic differentiation through Runx2 activation and, inversely, inhibits adipogenic differentiation through suppression of PPARγ activity. Thus, a functional correlation exists between TAZ activity and mechanical stress signals. YAP is also stimulated by extracellular matrix stiffness [1] and regulates Runx2-mediated gene transcription [36], although YAP suppresses Runx2-mediated gene transcription in certain contexts [37]. In this study, we did not investigate the role of YAP, but it would be interesting to study the functional role of YAP in shear stress-induced osteogenic differentiation.

TAZ functions as a transcriptional activator for osteogenic differentiation. It also stimulates cellular proliferation as an effector of the Hippo signaling pathway, which is involved in organ size control, cell proliferation, and differentiation. For functional activation, TAZ must translocate to the nucleus and interact with target transcription factors, including Runx2 and TEADs. Indeed, microfluidic-induced shear stress stimulates *CTGF* and *Cyr61*, which are TEAD target genes (Figure 3C). Nuclear localization of TAZ involves dephosphorylation at serine 89 because phosphorylated serine 89 interacts with 14-3-3 proteins, which sequester TAZ in the cytosol [3]. Thus, whether the phosphorylation status of TAZ is regulated by shear stress is an intriguing question.

Interstitial flow produces a lower shear stress than blood flow [15]. Under physiological conditions, MSCs may receive interstitial shear stress of the order of 0.01 Pa to 0.1 Pa, whereas endothelial cells in arteries may experience even over 1 Pa of shear stress [38]–[40]. Our microfluidic chip generated a physiological level of fluidal shear stress continuously without a peripheral device in the culture chamber. This allowed us to mimic the *in vivo* environment easily and facilitated the *in vitro* study of cellular functions mediated by interstitial fluid-induced stress.

Shear stress affects diverse biological functions, including embryonic development [15], [29], [41]. Commonly, fluidal shear stress stimulates the expression of several morphogenic response genes. Thus, our results suggest that our chip can be used to study *in vitro* the mechanisms involved in shear stress-induced embryonic development. To this end, it is notable that TAZ is involved in the self-renewal of embryonic stem cells and inhibits neuroectodermal lineage development. Thus, studying the lineage development of embryonic stem cell with our chips would be interesting.

In this study, we observed that shear stress activates TAZ through Rho signaling pathway, but detailed mechanisms were not addressed. Because Rho mediated actin polymerization is critical for TAZ activation, it seems that certain polymerization driven actin signaling components affect TAZ activity. It might directly regulate TAZ expression and localization, or indirectly regulate Hippo signaling components. In either way, further investigation is required for understanding the mechanism.

## Conclusions

We used a microfluidic system, in which a physiological level of low shear stress was induced by osmotic pressure, to investigate shear stress-induced osteogenic differentiation related to TAZ activity. Shear stress increased TAZ expression and nuclear localization and thus facilitated expression of osteoblastic marker genes. Shear stress-induced Rho activation was required for the nuclear localization of TAZ. Thus, we showed that TAZ is an important signaling mediator in interstitial fluid-driven shear stress-induced osteogenesis.

## Supporting Information

Figure S1 Replica of Figure 2B with velocity distribution. Each inset image of the channel is exactly the same as Figure 2B, from left to right, respectively. Wall shear stress distributions show the computed values on the bottom wall (at height  =  0 μm), where cells are seeded, but the velocity distribution (small arrows) are the computed values on the mid-plane (at height  =  100 μm) of the channel (total height  =  200 μm) since the velocity on the bottom wall is zero due to no-slip boundary condition applied.
(TIF)

Figure S2 Shear stress stimulates bone mineralization. MSCs were loaded onto the chips and differentiated into osteoblasts in the presence of osteogenic differentiation media for 21 days. Osmotic pump was changed at every 6 days for long-term culture. Mineralization activity in the differentiated cells was assessed by Von Kossa staining. Control cells were incubated in the osteogenic differentiation media without low shear stress and their media was changed every day.
(TIF)
