# Supplementary figures and images for: Shear Stress Induced by an Interstitial Level of Slow Flow Increases the Osteogenic Differentiation of Mesenchymal Stem Cells through TAZ Activation
Source: PLoS One. 2014 Mar 21;9(3):e92427. doi: 10.1371/journal.pone.0092427 (PMC3962409; doi:10.1371/journal.pone.0092427)

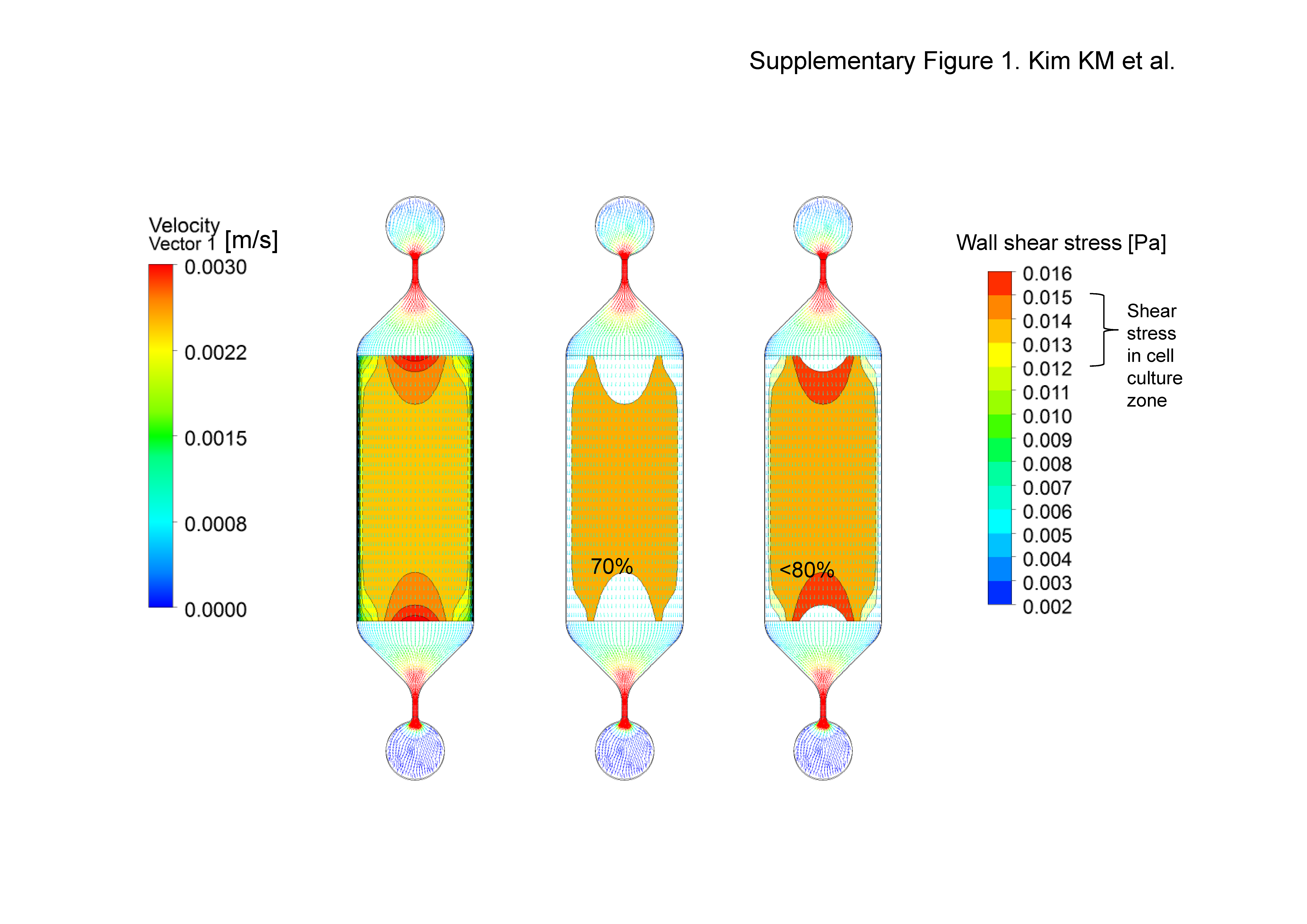

Supplement: Figure S1 — Replica of Figure 2B with velocity distribution. Each inset image of the channel is exactly the same as Figure 2B, from left to right, respectively. Wall shear stress distributions show the computed values on the bottom wall (at height = 0 μm), where cells are seeded, but the velocity distribution (small arrows) are the computed values on the mid-plane (at height = 100 μm) of the channel (total height = 200 μm) since the velocity on the bottom wall is zero due to no-slip boundary condition applied. (TIF) [file pone.0092427.s001.tif]

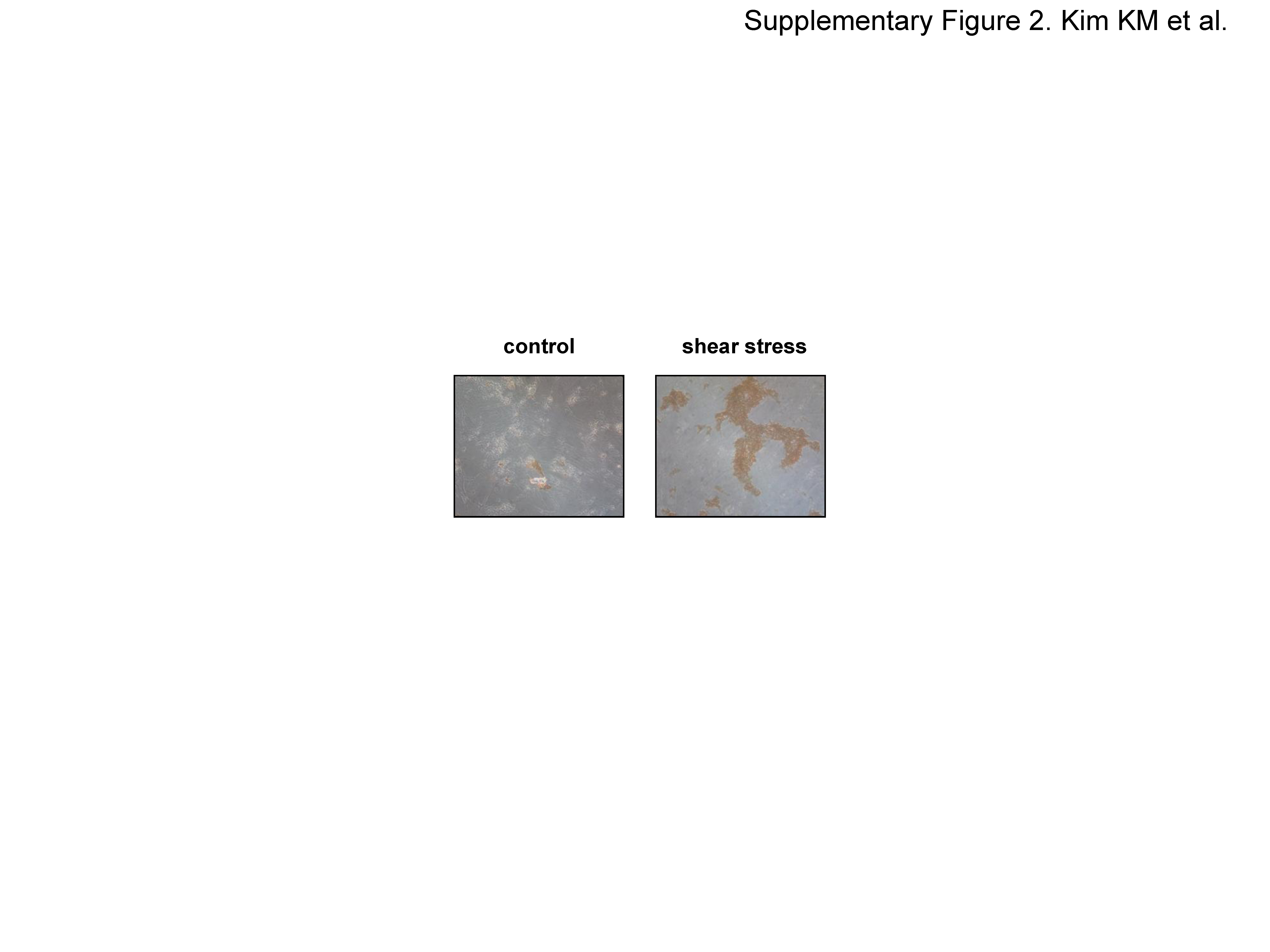

Supplement: Figure S2 — Shear stress stimulates bone mineralization. MSCs were loaded onto the chips and differentiated into osteoblasts in the presence of osteogenic differentiation media for 21 days. Osmotic pump was changed at every 6 days for long-term culture. Mineralization activity in the differentiated cells was assessed by Von Kossa staining. Control cells were incubated in the osteogenic differentiation media without low shear stress and their media was changed every day. (TIF) [file pone.0092427.s002.tif]
